# Small molecule inhibitors block Gas6-inducible TAM activation and tumorigenicity

**DOI:** 10.1038/srep43908

**Published:** 2017-03-08

**Authors:** Stanley G. Kimani, Sushil Kumar, Nitu Bansal, Kamalendra Singh, Vladyslav Kholodovych, Thomas Comollo, Youyi Peng, Sergei V. Kotenko, Stefan G. Sarafianos, Joseph R. Bertino, William J. Welsh, Raymond B. Birge

**Affiliations:** 1 Rutgers University, New Jersey Medical School, Department of Microbiology, Biochemistry and Molecular Genetics, Cancer Center, 205 South Orange Ave, Newark, NJ 07103, USA; 2 Rutgers University, Cancer Institute of New Jersey, 195 Little Albany Street, New Brunswick, NJ 08903, USA; 3 Department of Molecular Microbiology and Immunology, and Department of Biochemistry, Bond Life Sciences Center, University of Missouri, Columbia, MO 65211, USA; 4 Rutgers University, Office of Advanced Research Computing, 96 Frelinghuysen Road, Piscataway, NJ 08854, USA; 5 Rutgers University, Robert Wood Johnson Medical Center, Department of Pharmacology, 675 Hoes Lane, Piscataway, NJ 08854, USA

## Abstract

TAM receptors (Tyro-3, Axl, and Mertk) are a family of three homologous type I receptor tyrosine kinases that are implicated in several human malignancies. Overexpression of TAMs and their major ligand Growth arrest-specific factor 6 (Gas6) is associated with more aggressive staging of cancers, poorer predicted patient survival, acquired drug resistance and metastasis. Here we describe small molecule inhibitors (RU-301 and RU-302) that target the extracellular domain of Axl at the interface of the Ig-1 ectodomain of Axl and the Lg-1 of Gas6. These inhibitors effectively block Gas6-inducible Axl receptor activation with low micromolar IC_50s_ in cell-based reporter assays, inhibit Gas6-inducible motility in Axl-expressing cell lines, and suppress H1299 lung cancer tumor growth in a mouse xenograft NOD-SCIDγ model. Furthermore, using homology models and biochemical verifications, we show that RU301 and 302 also inhibit Gas6 inducible activation of Mertk and Tyro3 suggesting they can act as pan-TAM inhibitors that block the interface between the TAM Ig1 ectodomain and the Gas6 Lg domain. Together, these observations establish that small molecules that bind to the interface between TAM Ig1 domain and Gas6 Lg1 domain can inhibit TAM activation, and support the further development of small molecule Gas6-TAM interaction inhibitors as a novel class of cancer therapeutics.

The TAM receptors (Tyro-3, Axl, and Mertk) are a family of three homologous type I receptor tyrosine kinases (RTKs) that have important roles in homeostasis and the resolution of inflammation under physiological conditions. Pathophysiologically, TAMs are frequently overexpressed in a wide variety of human cancers that are associated with tumor progression and resistance to targeted therapeutics. Structurally, TAMs share a highly conserved intracellular kinase domain and a less conserved extracellular region characterized by two tandem immunoglobulin-like (Ig) domains and two tandem Fibronectin type III repeats^1^,^2^,^3^. The major ligands for TAMs are the vitamin K-dependent soluble proteins, Growth arrest-specific factor 6 (Gas6) and Protein S (Pros1), which interact with the tandem Ig1 (major contact) and Ig2 (minor contact) domains to trigger receptor dimerization and activation^4^. The three-dimensional structure of the Axl Ig1/Ig2 duet, in complex with Gas6 Laminin- G like domains (Lg1/2), has been solved by X-ray crystallography at 3.3 Å resolution (RCSB PDB entry 2C5D), confirming the main features of the ligand-receptor interface necessary for high-affinity ligand binding^5^.

Functionally, TAM receptors are not essential for embryonic development whereby triple knockout mice of Tyro-3, Axl, and Mertk have surprisingly unremarkable phenotypes through early postnatal life. However, in adulthood, particularly after puberty, persistent triple TAM knockout mice develop systemic chronic inflammation characterized, in part, by the loss of negative regulation of toll-like receptors (TLR) receptors in myeloid-derived cells^6^,^7^, inability to clear apoptotic cells (by a process known as efferocytosis^8^), in peripheral tissues, and constitutive elevation in pro-inflammatory cytokines that drive age-dependent autoimmunity^9^. These studies demonstrated that TAMs are not essential kinases, but rather have specialized functions as homeostatic receptors that participate in the clearance of apoptotic cells and the resolution of inflammation (reviewed^1^,^10^). Single knockouts of Tyro-3, Axl, or Mertk share some of the aforementioned effects of enhanced inflammation and hyper-activation of immune subsets, albeit with milder phenotypic outcomes, due in part to the non-overlapping expression of TAMs in different immune subsets such as macrophages (M1 versus M2), dendritic cells (DCs), and Natural Killer cells (NK cells)^11^. The lack of overt pathology in the TAM knockout mice implies that acute TAM therapeutics are unlikely to incur serious side effects by inhibiting essential biological functions.

All three TAM receptors are overexpressed in a wide spectrum of human cancers, and clinically associated with aggressive tumor grade and poor survival outcome (reviewed in^1^). For example, overexpression of TAMs can drive conventional oncogenic signaling and survival pathways in both hematopoietic and solid cancers^12^,^13^, epithelial to mesenchymal transition (EMT), and metastasis^14^. Gas6 is also concomitantly overexpressed in many cancers^1^. In addition, induction of TAM expression offers an escape mechanism for tumors that have been treated with oncogene-targeted agents including acquired resistance to EGFR^15^,^16^,^17^,^18^,^19^, PI3Kα^20^, FLT3^21^ and ALK^22^ targeted inhibitors, chemo-resistance^23^,^24^,^25^ and radiotherapy resistance^26^. Equally important, TAMs (particularly Axl and Mertk) are expressed on tumor-infiltrating myeloid-derived cells such as macrophages, DCs, and NK cells and have been identified as suppressors of anti-tumor immunity^6^,^27^,^28^. Indeed, inhibition of TAM expression/function, either via genetic ablation or via targeted TKI-based therapeutics, improves overall tumor immunity^29^,^30^, suggesting that TAMs may act as immune checkpoint inhibitors akin to CTLA-4, PD-1, and PD-L1.

Coincident with clinical evidence linking TAMs with poor patient outcomes in cancer, there has been great interest in recent years to develop TAM therapeutics in the form of small molecule tyrosine kinase inhibitors (TKIs,) antagonistic monoclonal antibodies (mAbs), and fusion proteins (Axl-Fc) that act as decoy receptor traps to neutralize TAM ligands, each of which has distinct modes of action and specific strengths and weaknesses. Here we describe a unique approach to inhibit TAM receptors by novel small molecule inhibitors that block binding of the Lg domain in Gas6 to the major Ig1 domain in the TAM extracellular region. Employing methods in rational (computer-aided) drug design, we discovered a series of lead compounds, exemplified by RU-301 and RU-302 that inhibit Axl reporter cell lines and native TAM receptors cancer cell lines with low-micromolar IC_50s_. Furthermore, using homology models and biochemical verifications, we show that RU301 and 302 also inhibit Gas6 inducible activation of Mertk and Tyro3 suggesting they can act as pan-TAM inhibitors that block the interface the between TAM Ig1 ectodomain and Gas6 Lg domain. These observations suggest that in addition to TKIs, therapeutic mAbs, and decoy receptors, small molecule extracellular inhibitors of the TAM Ig1 domain/Gas6 interface may represent an important strategy for developing TAM therapeutics.

## Results

### Identification of inhibitor binding sites in the Axl Ig1-Gas6 interface

**Figure 1:**
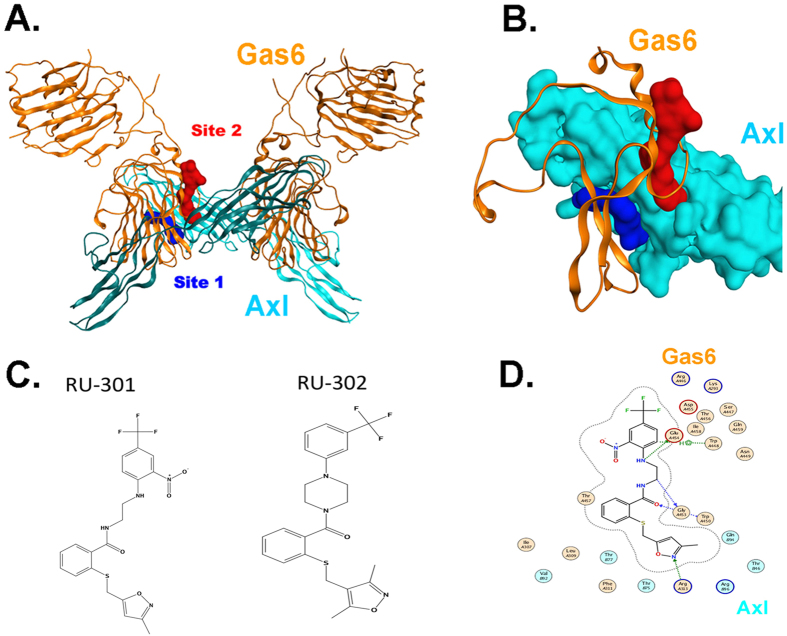
Molecular representation of drug targetable region in Axl-Gas6 complex, from RCSB PDB-2C5D. (**A**) Ribbon model depicting the targetable interfaces (in space filling) between Gas6 (orange) and Axl-Ig1 (cyan), with magnification of two targetable sites: Site 1 (blue space filling) and Site 2 (red space filling). **(B)** Inset of the detailed targetable interfaces between Gas6 and Axl from panel A. **(C)** Structures of RU-301 and RU-302 **(D)** Putative docking pose of RU-301 in Site 2, lined by contact residues in Gas6 (orange) and Axl (cyan).

The structure of the extracellular domain of Axl in a protein-protein complex with its endogenous extracellular ligand Gas6 has been solved by X-ray crystallography at 3.3 Å resolution^5^. Two distinct regions critical for Gas6 binding to Axl Ig1 were identified as putative druggable sites (Fig. 1A,B). Site 1 (S1) juxtaposes Glu59 in Axl and Arg310 and Lys312 in Gas6 that engage in strong electrostatic interactions; this coupling has been cited as key to Gas6 binding to Axl^5^. Structural alignment of the available X-ray crystal structures of Axl and Tyro-3 together with a homology model of Mertk suggests that the S1 site is unique to Axl and not present in Tyro-3 or Mertk (data not shown).

Site 2 (S2) is located in the hydrophobic interface between Gas6 and the Ig1 domain of Axl, with a significant portion extending into Gas6, the major Lg1 modular domain (Fig. 1A,B), suggesting that inhibitors that occupy this region may interfere with Gas6 binding and TAM receptor activation. This site is near the major Axl/Gas6 contact region depicted in the X-ray crystal structure which is lined by residues Thr46, Thr75, Thr77, Val92, Gln94 and Arg96 in Axl, and mostly apolar residues from the region 446–459 as well as Ile307, Leu309, Phe311, Thr461, Met468 in Gas6^5^ (Fig. 1D). While Tyro-3, Axl, and Mertk share only 36% overall identity in their Ig1 and Ig2 domains (regions that bind Gas6)^12^, three TAMs share common topological motifs in this region. Indeed, superimposition of the available X-ray structures of Axl (PDB: 2C5D) and Tyro-3 (PDB: 1RHF) reveals that the overall 3D structural organization and shape of Ig1 domains of both receptors are quite similar. Although corresponding structural information for Mertk in the Ig1/Ig2 domains is unavailable, homology modeling of the Mertk Ig1 and Ig2 domains (using Axl and Tyro-3 as templates) also suggests overall similarity in the Gas6 interaction regions, including conservation of a hydrophobic region that extends to the Gas6 Lg1 domain (Suppl. Fig. 1). Such structural comparisons offer clues that RU-301 and RU-302 small molecule inhibitors may exert pan-TAM inhibitory activity by binding at the interface between Gas6 and the Ig1 domain of the respective TAMs. Furthermore, the notion of a pan-TAM inhibitor would present opportunities to address a broad range of pathologies mediated by TAM receptor function including cancer^12^.

### Inhibitors target the TAM Ig1-Gas6 interface and block Gas6-dependent TAM activation

**Figure 2:**
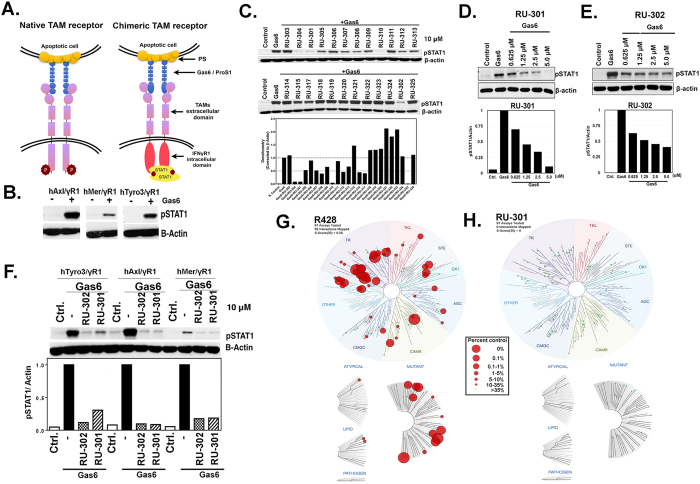
Lead compounds RU-301 and RU-302 show inhibitory activity in blocking TAM signaling. (**A**) Schematic representation of wild-type and chimeric TAM receptors. **(B)** Activation of the TAM-IFNγR1 chimeric receptors with Gas6 with pSTAT1 phosphorylation as readout for TAM activation. **(C)** Summary of small molecule inhibitors (10 μM) screening using TAM-IFNγR1 chimeric assay system showing RU-301 and RU-302 as the lead compounds. **(D)** Dose-response inhibition of Gas6-induced receptor activation by RU-301 (0.625–5.0 μM) using Axl-IFNγR1 cell lines. **(E)** Dose-response inhibition of Gas6-induced receptor activation by RU-302 (0.625–5.0 μM) using Axl-IFNγR1 cell lines. **(F)** Comparative inhibition of Gas6-induced receptor activation by 10.0 μM RU-301 and RU-302 on TAMs using TAM-IFNγR1 cell lines. **(G,H)** Comparison of R428 (a known Axl kinase domain targeting inhibitor) and RU-301 (Axl/Gas6 interaction inhibitor) by *KinomeScan*™ profiling for kinase domain interactions in the human kinome. Red circles represent drug binding kinases and the diameter of the circles represent the relative binding affinities.

Successive rounds of virtual screening of commercially available compound libraries, drug-receptor molecular docking at S2, and *in vitro* biological evaluation yielded a subset of potent inhibitor molecules with low micromolar inhibitor activity (see Materials and Methods). After 3 iterative cycles of testing compounds on chimeric human Tyro3, Axl or Mer -IFNγR1 reporter cell lines as a functional readout assay several lead compounds were further tested (Fig. 2A,B)^31^. As previously reported, hAxl/IFN-γR1, hTyro3/IFN-γR1 and hMertk/IFN-γR1 CHO cells stably express a chimeric receptor encoding Axl, Tyro-3 or Mertk-extracellular and trans-membrane domain and the intracellular domain of human IFN-γR1 (Fig. 2A). Activation of the chimeric receptor with Gas6 leads to chimeric receptor dimerization and subsequent pSTAT1 phosphorylation as a surrogate readout for TAM activation (Fig. 2B). Using the hAxl/IFN-γR1 reporter assay, both RU-301 and RU-302 exhibited highly potent inhibitory activity at 10 μM (Fig. 2C), and could be titrated to low-micromolar concentrations (Fig. 2D,E). These inhibitors partially inhibited binding between sAxl and full length Gas6 in pull-down assay (Suppl. Fig. 2).

To investigate whether RU-301 and RU-302 were indeed pan-TAM inhibitors, they were tested using hTyro3-IFNγR1 and hMertk-IFNγR1 cell lines in replicate experiments side-by-side with hAxl-IFNγR1. Consistent with this idea, similar inhibitory activity was observed when Mertk-IFNγR1 and Tyro3-IFNγR1 cells were treated with 10 μM RU-301 or RU-302 (Fig. 2F). Taken together, these data establish proof-of-principle that these lead compounds can effectively inhibit Gas6-induced activation of TAMs through disruption of the TAM-Gas6 interface in the extracellular region, providing a versatile alternative strategy to tyrosine kinase inhibitors (TKIs) and protein-based therapeutic modalities.

To determine whether these unique lead compounds have distinct activities to inhibitors that target the tyrosine kinase domain of Axl (TKIs), RU-301 was compared with R428, a well-characterized Axl inhibitor that has been developed for Axl-expressing tumors including metastatic breast cancer^32^ and NSCLC^33^. As shown in Fig. 2G,H, R428 bound many kinase domains in the human kinome, including Axl, whereas RU-301 showed drastically low and insignificant binding affinity (<35%) towards any of the kinases in the panel (Suppl. Table 1). In addition to the binding of Axl, R428 also bound to several other kinases, including Abl, Aurora kinases, KIT, PDGFR A and B, RET, TIE2, VEGFR2, JAK2, JAK3 and FLT3 kinases. As predicted, these data suggest that while RU-301 can inhibit Gas6-induced activation of TAM receptors, it does so independently of acting as a conventional TKI.

### Lead compounds inhibit native TAMs activation in native cancer cell lines

**Figure 3:**
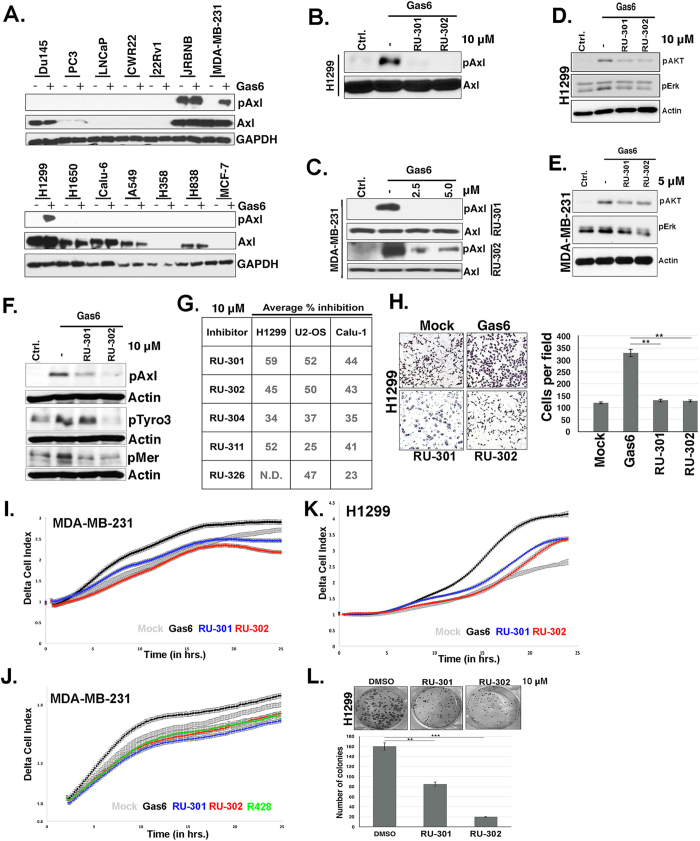
RU-301 and RU-302 inhibitors block the transforming potential of Gas6-induced endogenous TAM-dependent signaling. **(A)** Immunoblot screening of human cancer cell lines for inducible native Axl signaling by exogenous Gas6 treatment. **(B,C)** RU-301 and RU-302 inhibition of Gas6-induced Axl signaling in H1299 at 10.0 μM **(B)** and MDA-MB-231 at 5.0 μM **(C)** cells. (**D,E**) Immunoblot analysis of the effects of RU-301 and RU-302 on Gas6-mediated pAkt and pERK induction in H1299 at 10.0 μM **(D)** and MDA-MB-231 at 5.0 μM **(E)** cells. **(F)** The inhibitory effect of RU-301 and RU-302 on Gas6-induced phosphorylation of TAMs in H1299 cells at 10.0 μM. **(G)** Summary of percent inhibition of Gas6-induced receptor activation by 10.0 μM RU compounds on H1299, U2-OS and Calu-1 cells. **(H)** Transwell migration assay to test the efficacy of 10.0 μM RU-301 and RU-302 on Gas6-induced H1299 cells migration. **(I–K)** *xCELLigence*™ assay to measure the effect of 10.0 μM RU-301, RU-302 and R428 on real-time cell migration in MDA-MB-231 **(I,J)** and H1299 **(K)** cells. **(L)** Colony formation assay to estimate the effect of 10.0 μM RU-301 and RU-302 on clonogenic growth potential of Gas6 induced Axl activation in H1299 cells.

To translate results obtained from chimeric TAM receptor cell lines into the biology of native TAM receptor activation and more so focusing on Axl receptor, an array of cell lines were screened initially to identify cancer cell lines that (i) overexpressed Axl, and (ii) responded in an inducible manner to exogenous Gas6 (Fig. 3A). Consistent with previous reports, we identified some cell lines that had constitutive Axl activation (*i.e.* JRMNB) as well as other cell lines that had Gas6-inducible Axl activation, most notably the triple-negative breast adenocarcinoma MDA-MB-231 and the lung adenocarcinoma H1299 (Fig. 3A). Consistent with the inhibition of Gas6-mediated activation of Axl- IFNγR1 reporter lines, pre-treatment of H1299 cells with 10.0 μM RU-301 or RU-302 (Fig. 3B), 1 and 10 μM RU-301 or RU-302 (Suppl. Fig. 3) or MDA-MB-231 cells with 2.5 and 5 μM RU-301 or RU-302 (Fig. 3C) suppressed Gas6-inducible native phosphorylation of native Axl. Additionally, 10 μM RU-301 or RU-302 partially blocked Gas6-induced activation of Akt and Erk, well-known downstream targets of Axl in H1299 (Fig. 3D) or MDA-MB-231 at 5 μM (Fig. 3E). However, it is notable that H1299 and MDA-MB-231 cells harbor N-RAS and K-RAS mutations respectively, which may explain the partial effect of RU-301 and RU-302 inhibitors on these parameters. Using TAM-IFNγR1 reporter cell lines, we had established RU-301 and RU-302 to be pan-TAM inhibitors (Fig. 2F), and consistent with this observation, RU-301 and RU-302 inhibited the Gas6-induced phosphorylation of not only native Axl but also native Tyro3 and MerTK in H1299 at 10 μM (Fig. 3F), a cell line that contains all the three TAMs^34^. Similar inhibitory effects of Gas6-induced phosphorylation of Axl by 10 μM RU-301 and RU-302 were also observed in U2-OS and Calu-1 cells that express native Axl and are dependent on Gas6 for activation (percent inhibition is shown in Fig. 3G).

To assess functional outcomes, H1299 cells were pretreated with RU-301 or RU-302, after which oncogenic features (motility and clonogenic growth) were monitored as endpoints. As shown in Fig. 3H–L, 10 μM of both RU-301 and RU-302 strongly suppressed Gas6-inducible motility of H1299 lung cancer cell line through a 8 μm pore (using either standard Boyden chambers to measure migration endpoints (Fig. 3H) or real-time *xCELLigence*™ to measure early kinetics of migration) (Fig. 3K and Suppl. Fig. 4) as well as suppressed clonogenic growth of H1299 cells at 10 μM when cultured in the presence of Gas6 (Fig. 3L). Similar inhibitory effect on Gas6-induced cell migration of MDA-MB-231 cells were also observed in *xCELLigence*™ assays (Fig. 3I). Notably, the observed effects with RU-301 and RU-302 to block cell migration were as effective as R428 on MDA-MB-231 cells (Fig. 3J), a TKI and sAxl Ig1/Ig2 fusion protein on H1299 cells (Suppl. Fig. 4), a biologic that sequesters Gas6 away from the native Axl receptor.

### Lead compounds inhibit tumor growth in lung cancer xenograft model

**Figure 4:**
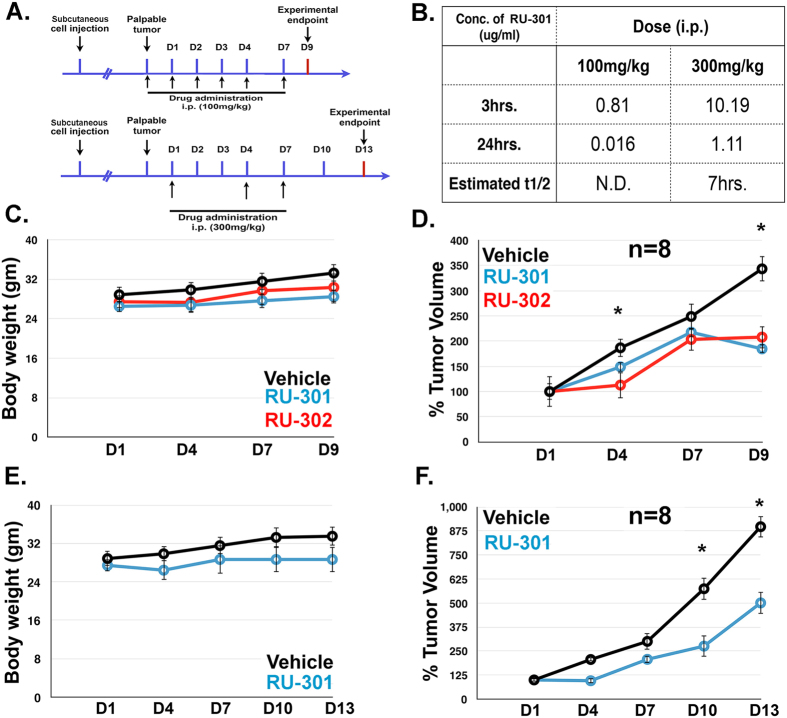
RU-301 and RU-302 suppress TAM mediated tumorigenicity in mice. (**A**) Schematics of the experimental design to assess the effect of lead compounds RU-301 and RU-302 on TAM mediated tumorigenicity by subcutaneous injection with 5 × 10^5^ human H1299 cells. (**B**) Pharmacokinetic profiles of RU-301 following intraperitoneal injection of 100 and 300 mg/kg to mice. **(C)** Body weight measurements of tumor-bearing mice upon administration with 100 mg/kg RU-301 or RU-302. **(D)** Suppression of tumor growth upon treatment with 100 mg/kg RU-301 or RU-302. **(E)** Body weight measurements upon treatment with 300 mg/kg RU-301. **(F)** Suppression of tumor growth upon treatment with 300 mg/kg RU-301. Error bars represent standard error of mean. *p* value indicated by *is <0.05.

Based on the promising *in vitro* and cell culture experiments showing anti-Axl activity of the lead compounds, their *in vivo* efficacies were evaluated using a murine NOD SCIDγ/human H1299 lung cancer xenograft model (Fig. 4). As described in the Methods section, SCID mice were subcutaneously injected with 5 × 10^5^ human H1299 cells in the hind flanks until palpable tumors were present (Fig. 4A). Subsequently, mice were injected daily with vehicle, RU-301 or RU-302 as shown in Fig. 4A. RU-301 and RU-302 significantly decreased tumor volume (Fig. 4D,F), while body weights were not significantly different at both 100 mg/kg and 300 mg/kg (Fig. 4C,E). Neither compound showed notable toxicity but both displayed good bioavailability with a t1/2 life of ~7–8 hours (Fig. 4B). Phospho Axl and phospho Mertk levels in the primary tumors were assessed by Western blotting but could not be detected (data not shown). Taken together, these studies support the further development of extracellular Gas6/TAM Ig1 inhibitors as anti-cancer therapeutics.

## Discussion

In this study, we have developed and characterized novel first-in-class Gas6/TAM receptor antagonists that interfere with the binding of Gas6 to the Ig1 domain of TAMs in the extracellular domain. These atypical TAM inhibitors show promising therapeutic potential, and can block TAM activation and signaling at low-micromolar concentrations. Moreover, preclinical studies presented here support their utility in both cell lines and *in vivo*, and indicate they have minimal toxicity and good bioavailability when administered to mice. Our studies provide a rationale that in addition to the continued development of tyrosine kinase inhibitors, therapeutic mAbs, and TAM-Fc extracellular decoy receptors as TAM therapeutics, a compelling alternative strategy exists that employs small molecule inhibitors that block the major groove between the TAM Ig1 domain and the Gas6 Lg1 domain in the extracellular domain.

In recent years, overexpression of TAM receptors and Gas6 has been reported in a wide range of human cancers, an axis that is associated with aggressive cancer phenotypes, emergence of drug resistance, immune escape, and overall poor patient survival. In tumor cells, activation of Axl has been associated with downstream signaling of proliferation and survival pathways such as Erk and Akt^35^,^36^,^37^,^38^, emergence of drug resistance via the direct phosphorylation of other tyrosine kinases such as MET and EGFR^23^,^39^, and epithelial to mesenchymal transition (EMT) via the up-regulation of TWIST and SLUG^14^,^40^,^41^. Notably, Gas6, the major ligand for Mertk and Axl, is also concomitantly overexpressed in the tumor microenvironment, establishing an autocrine loop that constitutively activate TAMs on tumor cells^42^,^43^.

Besides the intrinsic activation of TAMs in cancer, TAMs also contribute to tumor progression via their expression on infiltrating myeloid-suppressor cells, macrophages, and NK cells where they affect immune escape. For example, the infiltration of Mertk-expressing NK and M2 macrophages is associated with suppression of anti-tumor immune responses^27^,^29^. This places TAMs as unique oncogenic proteins that besides activating conventional oncogenic pathways alluded to above, also contribute to tumorigenesis in an unconventional manner by blocking signals necessary for immunogenic death and thereby promoting tolerance and immune suppression in the tumor microenvironment^6^. As such, generalized antagonists that act as pan-TAM inhibitors and target the extracellular binding site between Gas6 and TAMs may have opportunistic pleiotropic paracrine effects for multiple effector cells that coordinately improve outcomes in cancer patients. Further studies that investigate TAM/Gas6 interaction inhibitors in immune-competent mouse models will shed more light on these queries, particularly whether and how these drugs induce immunogenic anti-tumor responses when combined with therapeutics such as anthracyclines that induce immunogenic death.

As alluded to above, until now three major types of TAM inhibitors have been considered that include: (i) small molecule tyrosine kinase inhibitors (TKIs), (ii) antagonistic monoclonal antibodies, and (iii) TAM-Fc soluble decoy receptors^1^. All three approaches show therapeutic promise, and each has strengths and weaknesses. Small molecule TAM TKIs can show robust inhibitory activities *in vitro*, but most are not TAM specific, and show considerable off-target profiles^44^. For example, the Axl specific TKI inhibitor BGB324 (formerly known as R428) has a reported IC_50_ activity of 14 nM for Axl (compared with 700 nM and 1400 nM IC_50s_ for Mertk and Tyro-3)^32^ but BGB324 also has off-target effects on VEGFR, Abl, Tie-2 and MET kinases (Fig. 2G). Likewise, UNC569 and UNC1666, which have preferred specificity for Mertk, compared to Axl and Tyro-3, also have off-target effects on Flt3 and RET^45^,^46^. TKIs also induce drug resistance arising from inevitable on-target mutations^47^,^48^. Finally, in contrast to TKIs that require entry into the cell before binding the receptor kinase-binding pocket, the subject inhibitors act in the extracellular domain at the interface between the Ig1 domain of Axl and the Lg1 domain of Gas6. This distinction may confer several advantages, such as lower risk of drug-induced resistance.

More recently, other promising strategies to target TAMs have focused on decoy receptor traps^49^ and antagonistic monoclonal antibodies^50^,^51^ for Axl and Mertk that are in pre-clinical development. In the case for the decoy receptors, elegant studies have engineered high affinity Axl decoy traps that bind and sequester Gas6 with femtomolar affinities^52^. These biologics show great promise as cancer therapeutics in aggressive pre-clinical models of human cancer with minimal overt toxicity, suggesting that targeting the Axl Ig1/Gas6 binding pocket is an attractive strategy for clinical developement. The novel class of small molecule inhibitors described here that target the Gas6/TAM interface in the extracellular domain may be conceptually similar to the Axl decoy receptors by preventing Gas6-mediated activation of TAMs. Current efforts aim to develop RU-301 and RU-302 derivatives with higher efficacy by rationale drug design.

Finally, whereas the focus of the current study is aimed at anti-cancer therapeutics, the development of TAM/Gas6 interaction inhibitors in this application will be directly relevant to other applications. First, the interaction of Gas6 with TAMs recently has been shown to have important consequences in arterial thrombosis formation^53^,^54^. Levels of Gas6 are elevated in thrombotic platelets and knockout of Gas6 blocks experimental thrombosis^55^,^56^. Inhibitors of Gas6 binding to TAMs are likely to benefit high-risk patients prone to platelet aggregation and thrombosis. Second, TAM inhibitors may also be therapeutically relevant as anti-viral antagonists. For example, HIV is an enveloped RNA virus that buds from the host plasma membrane in the latter stages of the infectious cycle. In doing so, HIV virus has high concentration of phosphatidylserine (PS) associated with the envelope, enabling HIV to act as an apoptotic cell mimic^57^,^58^,^59^. Binding of HIV to TAMs receptors on macrophages or DCs is expected to induce immune suppression and tolerance and TAM inhibitors could be used to stimulate immune responses in HIV in combination with inhibitors to block replication. Similar inhibitory activities may be achieved with other viruses such as Dengue^60^ and Zika^61^.

In summary, we have developed and characterized a series of small molecule TAM/Gas6 interaction inhibitors that target a binding site formed at the interface between the Ig1 domain of the receptor and the Gas6 Lg1 domain. Unlike other TAM inhibitors, these inhibitor compounds appear to act by blocking a protein: protein interaction formed at the TAM/Gas6 binding interface that is necessary for TAM receptor dimerization and activation.

## Materials and Methods:

### Computational methods

The X-ray crystal structure of the soluble Gas6/Axl complex (RCSB PDB entry 2C5D) was used to identify possible small molecule ligand binding sites. Four independent programs: (i) the Q-siteFinder^62^, (ii) the SiteMap^63^ (Schrödinger Suite, NY), (iii) the SiteID (Sybyl, Tripos Associates, St. Louis, MO) and SiteFinder (MOE, CCG, Montreal, Canada) were used for this purpose. All ligand binding sites were visually inspected for their suitability to bind drug-like compounds. For Q-siteFinder and SiteID, the crystal structure of Gas6/Axl complex was used without any modification, whereas for SiteMap, the structure was prepared by ‘Protein Preparation Wizard’ utility of Schrödinger Suite. The ‘Protein Preparation Wizard’ adds missing residues and hydrogen atoms, generates protonation states of certain amino acid residue and optimizes hydrogen atom conformation. Final structure was further subjected to 1000 iterations of energy minimization to relax the structure, using either the OPLS_2005 force field in the ‘Impact’ module of the Schrödinger Suite or the Amber10:EHT force field in MOE. The hit-to-lead scheme encompassed successive iterative rounds of ligand-based virtual screening, ligand-receptor docking and scoring, and *in vitro* biological evaluation in cell-based assays. Each iterative cycle enriched the quality of the top-scoring hits, which were further refined manually based on considerations of conformational flexibility, biophysical attributes, and constraints imposed by the receptor binding pocket. Virtual screening of commercially available drug-like compound collections found in ZINC^64^,^65^ was performed using MOE and Avalanche tools^66^. Top-ranking virtual hits were visually inspected and culled for further evaluation, and the remaining ~500 top hits were submitted to AutodockVina^67^ and MOE DOCK for subsequent docking and scoring. In each cycle, a subset of about 20 hits was selected from among the top-scoring 50 hits for subsequent biological evaluation in panels of cell-based inhibition assays. Enrichment was monitored in terms of various measures, including the overall inhibitory activity of the subset, the inhibitory activity of the most active hit in the subset, calculated biophysical features (e.g., Lipinski Ro5 parameters), and receptor docking scores. Typically, more than a single hit was selected from each panel for the next iterative cycle in order to retain favorable drug-like attributes.

### Gas6 conditioned medium and antibodies

Human Gas6 conditioned medium (CM) was obtained from stable HEK293 cells secreting human Gas6 into serum-free media in the presence of 10 μg/mL vitamin K1 (Hospira). After 72 hours, the Gas6 conditioned medium was collected, and validated for the presence of Gas6 γ-carboxylation (by immunoblot) and activity of Gas6 (via activation of pSTAT1 in hAxl/IFNγ-R1 cells). Antibodies used were as follows: anti-phosphorylated hAxl (Cell Signaling), hAxl (Santa Cruz), anti-phosphorylated STAT1 (BD Bioscience), anti-phosphorylated hAkt (Santa Cruz), anti-hGas6 (R&D), anti-γ-carboxylation (Sekisui Diagnostics), GAPDH (Millipore) and anti-β-Actin (Cell Signaling).

### Detection of activation of TAM Receptors

Stable hAxl/IFN-γR1 CHO reporter cell lines (contain human Axl extracellular domains and transmembrane and intracellular domains of human IFN-γR1), H1299, MDA-MB-231, CALU1 and U2-OS cells were serum starved and stimulated with human Gas6 CM ± small molecule inhibitors/test compounds for 30 min. Whole cell lysates were prepared using HNTG buffer (20 mM HEPES, pH 7.5, 150 mM NaCl, 10% glycerol, 1% Triton X-100, 1 mM PMSF, 1 mM Na_3_VO_4_, 10 mM Na_2_MoO_4_, 1 mM EDTA, 10 mM NaF and 20 μg/mL aprotinin). The resulting lysates were resolved on SDS-PAGE gel and immunoblotted with respective antibodies.

### Kinome scan profiling to assess kinase domain binding

The KINOMEscan profiling of the inhibitors were performed as per directions by the manufacturer (DiscoverX) against a panel of human protein kinases. Each small molecule inhibitor (R428 and RU-301) was screened against the panel of 97 protein kinases at a concentration of 10 μM to identify candidate kinase targets that bind to the respective inhibitors, and for each interaction observed in this primary screen a quantitative dissociation constant (Kd) was determined. Binding constants are correlated with primary screening results, where lower Percent Control (% Ctrl) values are associated with low Kd values (higher affinity interactions).

### Real-time cell migration assay using the RTCA DP xCELLigence™ system

The real-time cell migration assay using *xCELLigence*™ system was performed as previously described^68^. Briefly, post serum-starvation, H1299 or MDA-MB-231 cells were counted and 40,000 cells/100 μl were added in serum-free media in the top chamber of CIM plate, ±Gas6 ± 10 μM test compounds in triplicates. Media containing 10% FBS was added as a chemo-attractant in the lower chamber of CIM plate. Readings for changes in the cell index (CI) were taken every 10 min for 24 h and cell migration was represented as a change in cell index versus time.

### Colony forming assays

H1299 cells were plated in 6 well plates at a very low density (~ 250 cells/well) and allowed to adhere overnight. The cells were then treated with the test compounds as specified and incubated with the test compounds for 14 days. After 14 days, colonies were stained with 0.25% crystal violet in 95% ethanol. Colonies consisting of more than 50 cells were counted.

### *In vivo* mice xenograft studies

5 × 10^5^ H1299 cells in 100 μl of PBS were injected subcutaneously into the both flanks of 4–6 week NOD/SCIDγ mice. Once tumors were palpable, the mice were randomized into 3 groups wherein each group had 4 mice with tumors thus making n = 4 × 2 = 8 per group. Control mice were intra-peritoneally (i.p.) treated with vehicle (DMSO) and treatment groups with RU-301 (day1, day2, day3 and day4) (i.p.) at a dose of 100 mg/kg and 300 mg/kg, or RU-302 (day1, day2, day3 and day4) i.p. at a dose of 100 mg/kg. Tumor size and body weights were measured three times a week and the tumor volumes were calculated. Institutional Animal Care and Use Committee (IACUC) at Cancer Institute of New Jersey (CINJ) Rutgers University approved all protocols concerning animal use in this study. All the animal experiments were performed in accordance with relevant guidelines and regulations recommended by Rutgers University.

### Data analysis

Immunoblots were obtained within a linear range of exposure and intensities were quantified using ImageJ. All experiments were repeated at least three times and statistical analysis performed using GraphPad Prism. Descriptive statistics for quantitative variables were summarized using mean ± standard deviation. Differences between groups were tested by T-test or one-way ANOVA followed by Tukey post-hoc test. Differences with a P value of <0.05 were considered statistically significant.

## Additional Information

**How to cite this article**: Kimani, S. G. *et al*. Small molecule inhibitors block Gas6-inducible TAM activation and tumorigenicity. *Sci. Rep.* **7**, 43908; doi: 10.1038/srep43908 (2017).

**Publisher's note:** Springer Nature remains neutral with regard to jurisdictional claims in published maps and institutional affiliations.

## Supplementary Material

Supplementary Information
