# Supplementary material for: Small molecule inhibitors block Gas6-inducible TAM activation and tumorigenicity
Source: Sci Rep. 2017 Mar 8;7:43908. doi: 10.1038/srep43908 (PMC5341070; doi:10.1038/srep43908)
Supplement: Supplementary Information [file srep43908-s1.pdf]

Supplementary information for

Small molecule inhibitors block Gas6-inducible TAM activation and tumorigenicity

Stanley G. Kimani[1]*, Sushil Kumar[1]*, Nitu Bansal[2]*, Kamalendra Singh[3], Vladyslav Kholodovych[4,5], Thomas Comollo[1], Youyi Peng[2], Sergei V. Kotenko[1], Stefan G. Sarafianos[3], Joseph R. Bertino[2], William J. Welsh[2,5, #], and Raymond B. Birge[1#]

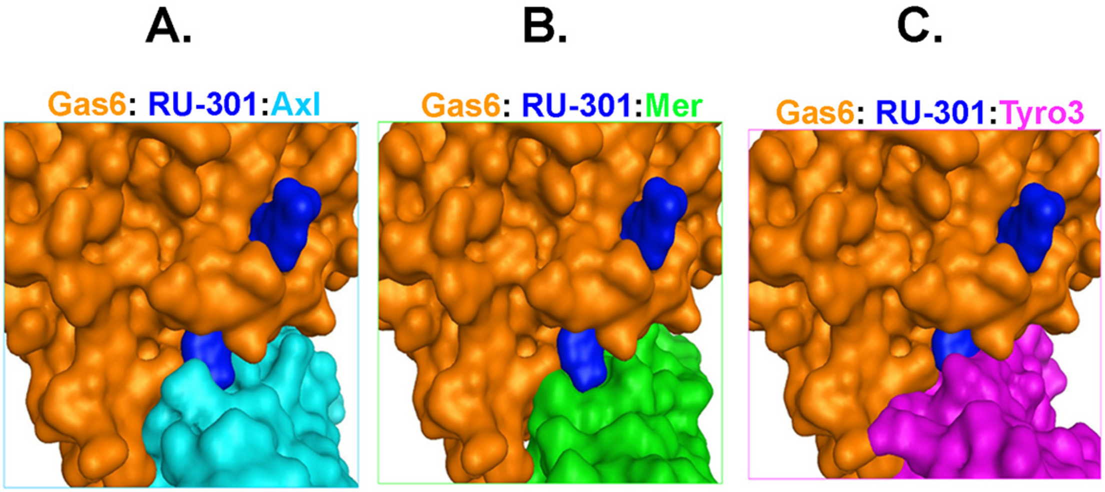

Supplementary Figure 1: Binding of RU-301 in a pocket located at the interface of Gas6 and TAM-Ig1, based on computational drug-receptor docking studies. The binding pocket consists predominantly of Gas6 residues, together with a small region of TAM residues that cradle one end of the drug. RU-301: blue; Gas6: orange; Axl: light teal (2C5D.pdb) **(A)**; Mertk: green (homology model based on Axl) **(B)**; Tyro-3: magenta (1RHF.pdb) **(C)**. All images are rendered from identical perspectives to facilitate comparison.

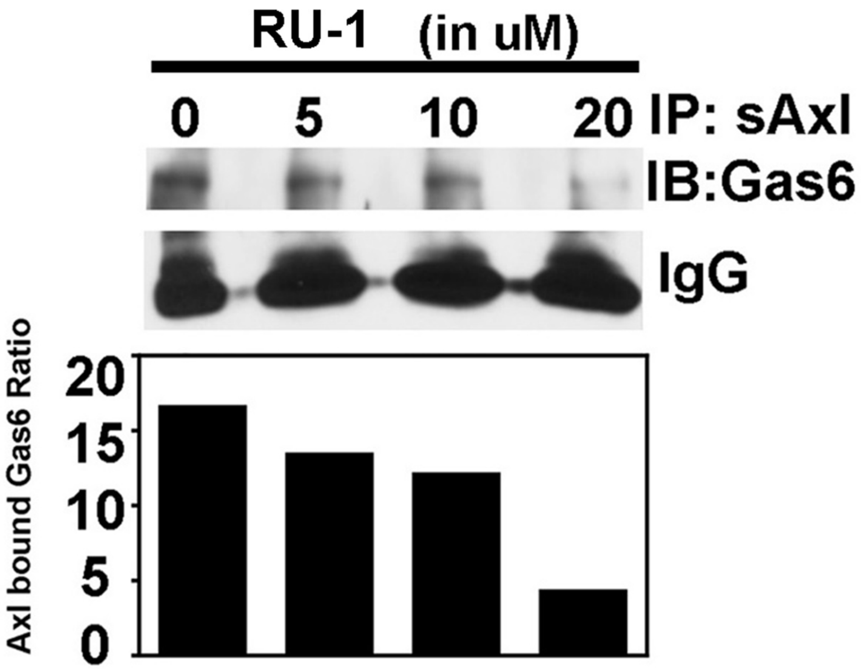

Supplementary Figure 2: Competitive binding assay to test the RU-1, parental inhibitor molecule of RU-301/2 as a protein-protein inhibitor between sAxl (contains Ig1 and Ig2) and Gas6. His tagged- sAxl were co-incubated with increasing amounts of a parental inhibitor molecule RU-1 in a 50 μl reaction mixture of Tris-NaCl Buffer and incubated at 37 °C for 60 min, with intermittent mixing. Thereafter, equal amounts of hGas6 were added to each tube and incubated at 37 °C for another 60 min, with intermittent mixing. The resulting reaction mix was immunoprecipitated by anti-His antibody and immunoblotted for sAxl- bound Gas6 after multiple washes with lysis buffer. RU-1 was able to partially compete Gas6-Axl binding using a pull-down binding assay when purified Gas6 (Amgen) was incubated with a baculovirus-produced purified His-tagged soluble human Axl Ig1/Ig2 recombinant protein (indicating these inhibitors as agents that target the interface of Gas6 and Ig1/ Ig2 domain of Axl).

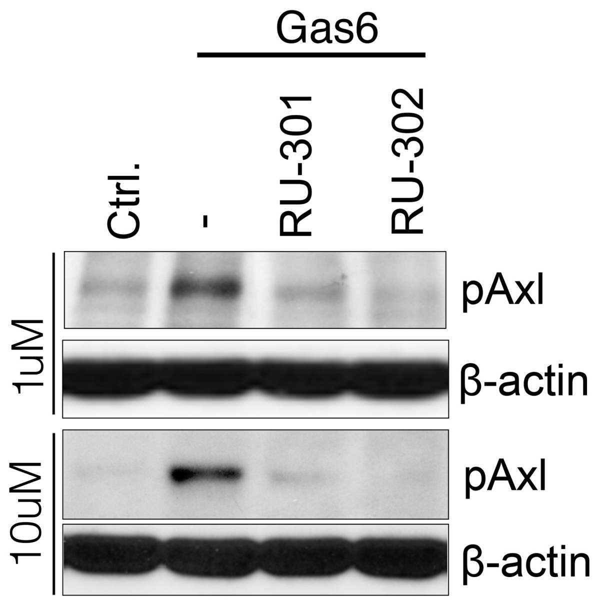

Supplementary Figure 3: Dose dependent inhibition of native Axl receptor by RU-301 and RU-302. Two different concentrations of RU-301 and RU-302 (1 and 10 μM) were used to pretreat the H1299 cells before and during Gas6 induction. The inhibition of the respective RU-301 and RU-302 concentration of Gas6-induced Axl signaling in H1299 cells was then assessed by immunoblot analysis and shows robust inhibition of Gas6 induced Axl activation by RU301 and RU302.

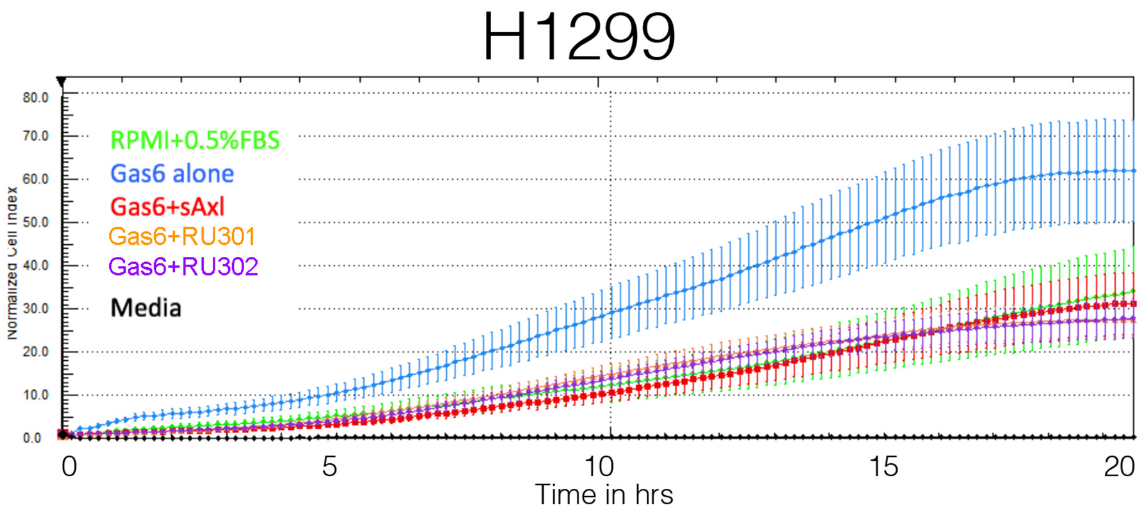

Supplementary Figure 4: Comparative inhibition of RU301 and RU302 with sAxl inhibitor for cell migration. sAxl and RU-301 and 302 were used to pretreat the H1299 cells before Gas6 induction. The resulting cells were then assessed for the comparative migration capability using xcelligence system. The results indicate the comparable and robust inhibition of cell migration by RU-301 and RU-302 with sAxl that is similar to the Gas6 untreated control.

Gene Symbol	R428 %Ctrl @ 10000nM	RU-301 %Ctrl @ 10000nM
ABL1(E255K)-phosphorylated	0.25	94
ABL1(T315I)-phosphorylated	0.9	100
ABL1-nonphosphorylated	0.25	100
ABL1-phosphorylated	0.1	81
ACVR1B	26	96
ADCK3	18	76
AKT1	100	88
AKT2	100	100
ALK	60	100
AURKA	0.15	89
AURKB	0.45	73
AXL	0.1	89
BMPR2	23	78
BRAF	64	99
BRAF(V600E)	70	94
BTK	31	100
CDK11	98	94
CDK2	71	100
CDK3	72	93
CDK7	23	85
CDK9	92	95
CHEK1	15	83
CSF1R	0.35	90
CSNK1D	98	94
CSNK1G2	4.6	100
DCAMKL1	98	81
DYRK1B	46	100
EGFR	74	100
EGFR(L858R)	10	94
EPHA2	71	96
ERBB2	46	94
ERBB4	34	83
ERK1	100	100
FAK	83	100
FGFR2	30	100
FGFR3	30	91
FLT3	0.1	99
GSK3B	74	98
IGF1R	99	98
IKK-alpha	0.5	86
IKK-beta	4.8	100
INSR	14	89
JAK2(JH1domain-catalytic)	0	87
JAK3(JH1domain-catalytic)	0.2	95
JNK1	14	91
JNK2	96	97
JNK3	46	100
KIT	0.1	96

Gene Symbol	R428 %Ctrl @ 10000nM	RU-301 %Ctrl @ 10000nM
KIT(D816V)	0.15	100
KIT(V559D,T670I)	0.05	85
LKB1	60	77
MAP3K4	0.7	100
MAPKAPK2	87	100
MARK3	49	87
MEK1	3	75
MEK2	0.2	71
MET	9.4	88
MKNK1	19	78
MKNK2	0	100
MLK1	2.2	100
p38-alpha	78	93
p38-beta	84	100
PAK1	74	97
PAK2	69	96
PAK4	85	93
PCTK1	90	100
PDGFRA	1.4	99
PDGFRB	0	91
PDPK1	34	99
PIK3C2B	31	100
PIK3CA	45	100
PIK3CG	21	100
PIM1	67	82
PIM2	100	83
PIM3	84	86
PKAC-alpha	48	90
PLK1	2.9	95
PLK3	29	91
PLK4	1.7	81
PRKCE	24	91
RAF1	100	84
RET	0	97
RIOK2	94	100
ROCK2	3	75
RSK2(Kin.Dom.1-N-terminal)	2.1	74
SNARK	0.65	100
SRC	14	96
SRPK3	3.8	80
TGFBR1	75	100
TIE2	0.45	100
TRKA	3.9	100
TSSK1B	22	78
TYK2(JH1domain-catalytic)	17	55
ULK2	1.6	97
VEGFR2	1	99
YANK3	100	100
ZAP70	33	76

$0 \leq x < .1$ | $.1 \leq x < 1$ | $1 \leq x < 10$ | $10 \leq x < 35$ | $x \geq 35$

Supplementary Table 1: Detailed quantitative results of screening RU-301 and R428 against 97 protein kinases. Each small molecule inhibitor was screened against the panel of 97 protein kinases at a concentration of 10μM to identify candidate kinase targets, and for each interaction observed in this primary screen a quantitative dissociation constant (Kd) was determined. Binding constants are correlated with primary screening results, where lower Percent Control (% Ctrl) values are associated with low Kd values (higher affinity interactions).

Supplementary methods:

Virtual Screening of Compounds:

Virtual screening of the compounds was conducted as follows. First, the virtual screening of the Maybridge Hitfinder library of drug-like compounds was conducted for their propensity to bind in a pocket near Gas6/Axl major interaction site. The molecular models of library compounds were generated from the 'structure data files' (sdf) using LigPrep, a ligand preparation tool interfaced with 'Maestro' molecular modeling program (Schrödinger Inc. NY). The structures generated by LigPrep (~16,000 total compounds) were docked into the selected site using the software 'Glide' (Schrödinger Suite, with extra precision (XP) option of Glide. We used the combination of Glide-score and visual inspection of all docked compounds and selected top 500 compounds for flexible docking using 'Induced Fit Docking' of Schrödinger Suite. The 'Induced Fit Docking' workflow allowed the optimization of side-chains of the binding pocket to filter the compounds for best binding energy. Top four compounds exhibiting best binding energy were selected for experimental validation and structure-activity relationship (SAR) studies.

Modeling of Ig domains of TAM receptors

TAM receptors share 72-75 % protein sequence similarity (54-59 % identity) within the intracellular region and are even more conserved inside the catalytic kinase domain. However, they vary much more within the extracellular region, with only 52-57 % of amino acid similarity (31-36 % identity). The availability of X-ray crystallographic data of the extracellular domain of the TAM receptors is sparse. The Protein Databank (PDB) contains two relevant structures, viz., the Ig like domain of human Axl complexed with Gas6 at 3.3 Å resolution (RCSB PDB 2C5D), and a fragment spanning the two N-terminal Ig domains of the extracellular part of human Tyro3 at 1.95 Å resolution (RCSB PDB 1RHF).

Structural alignment of the Ig domains of TAM receptors was based on the corresponding sequences of Ig1 and Ig2 domains of human Axl, Mertk and Tyro-3 receptors from The Universal Protein Resource (UniProt) entries hAXL: P30530 (Ig1: 27-128 and Ig2: 139-222); hMertk: Q12866 (Ig1: 81-186 and Ig2: 197-273) and hTyro-3: Q06418(Ig1: 41-128 and Ig2: 139-220). The structures of the respective Ig1 domains of Mertk and Tyro-3 were then each individually compared to the structure of the Ig1 domain of Axl at what was described as the "major binding site" for Gas6 for residues structurally homologous to interacting residues in Axl-Gas6 binding.

Axl and Tyro-3 Models - Chain D containing Axl's Ig1 and Ig2 domains was isolated from the Protein Data Bank entry 2C5D by deleting all other chains, ligands, water molecules, and ions, and chain A containing Tyro-3's Ig1 and Ig2 domains from PDB entry 1RHF was prepared in a similar fashion. The Tyro-3 structure then was structurally superposed on the Axl structure using protein backbone atoms in MOE.

Mertk Homology Model - The homology model of Mertk was created in MOE by using a D chain of Axl-Gas6 complex (2C5D) as a template and a sequence of Ig1 corresponding region of Mertk (Q12866:81-186) from UniProt. A tree-based method implemented in MOE with a BLOSUM62 scoring matrix was employed for alignment.
